# Monoethylhexyl Phthalate Elicits an Inflammatory Response in Adipocytes Characterized by Alterations in Lipid and Cytokine Pathways

**DOI:** 10.1289/EHP464

**Published:** 2016-07-06

**Authors:** Sara Manteiga, Kyongbum Lee

**Affiliations:** Department of Chemical and Biological Engineering, Tufts University, Medford, Massachusetts, USA

## Abstract

**Background:**

A growing body of evidence links endocrine-disrupting chemicals (EDCs) with obesity-related metabolic diseases. While it has been shown that EDCs can predispose individuals toward adiposity by affecting developmental processes, little is known about the chemicals’ effects on adult adipose tissue.

**Objectives:**

Our aim was to study the effects of low, physiologically relevant doses of EDCs on differentiated murine adipocytes.

**Methods:**

We combined metabolomics, proteomics, and gene expression analysis to characterize the effects of mono-ethylhexyl phthalate (MEHP) in differentiated adipocytes.

**Results:**

Repeated exposure to MEHP over several days led to changes in metabolite and enzyme levels indicating elevated lipogenesis and lipid oxidation. The chemical exposure also increased expression of major inflammatory cytokines, including chemotactic factors. Proteomic and gene expression analysis revealed significant alterations in pathways regulated by peroxisome proliferator activated receptor-γ (PPARγ). Inhibiting the nuclear receptor’s activity using a chemical antagonist abrogated not only the alterations in PPARγ-regulated metabolic pathways, but also the increases in cytokine expression.

**Conclusions:**

Our results show that MEHP can induce a pro-inflammatory state in differentiated adipocytes. This effect is at least partially mediated PPARγ.

**Citation:**

Manteiga S, Lee K. 2017. Monoethylhexyl phthalate elicits an inflammatory response in adipocytes characterized by alterations in lipid and cytokine pathways. Environ Health Perspect 125:615–622; http://dx.doi.org/10.1289/EHP464

## Introduction

Contamination of the environment with organic pollutants has emerged as a significant public health concern due to the pervasive nature of these contaminants. Of particular concern are endocrine-disrupting chemicals (EDCs), which comprise a structurally diverse group of chemicals that interfere with the endocrine system. Epidemiological studies have linked chronic EDC exposure to adverse effects on reproduction, development, and more recently, metabolic diseases. A growing number of studies have reported that perinatal exposure to certain EDCs, termed obesogens (Grün and Blumberg 2006), could contribute to weight gain through an adipogenic effect that leads to increased body fat mass. This hypothesis has gained support from both *in vivo* and *in vitro* studies. Progenitor cells isolated from the adipose tissue (AT) of mice exposed *in utero* to tributyltin (TBT) exhibit greater sensitivity towards adipogenic differentiation and increased basal expression of adipogenic differentiation marker genes (Kirchner et al. 2010). These and related findings have highlighted the potential for early-life EDC exposure to predispose the offspring toward an obese phenotype later in life by reprogramming stem cell fate, possibly through epigenetic changes.

Mechanistic information remains scant, however, for many other EDCs that are substantially more prevalent in the environment than TBT and have also been linked to obesity-related metabolic diseases. To date, studies have mainly focused on the impact of suspected obesogens on stem cell fate and tissue development, sometimes yielding conflicting results (Rubin et al. 2001; Ryan et al. 2010). Less attention has been paid to clarifying whether these chemicals can directly disrupt metabolic regulation in differentiated cells of adult tissue. In AT development, formation of new adipocytes via differentiation of progenitor cells is intimately coupled to the ensuing expansion of adipocytes (hypertrophy) via lipid accumulation; the enzymes and regulatory proteins responsible for lipid droplet (LD) formation are also markers of differentiation. In postadolescent humans, hypertrophy is the predominant mode of body fat mass increase, as the adipocyte turnover rate remains nearly constant at ~ 10% per year throughout adulthood (Spalding et al. 2008). Paradoxically, obese subjects exhibit a decreased capacity to form new lipid-storing adipocytes, which limits the overall plasticity of the AT (Danforth 2000) and pushes the mature adipocytes toward hypertrophic expansion in response to overfeeding.

Adipose cellular hypertrophy correlates with accumulation of pro-inflammatory immune cells in AT, which underpins insulin resistance and other metabolic dysfunctions associated with obesity-related diseases (Manteiga et al. 2013). It is possible that EDCs interfere with endogenous regulatory pathways to promote an inflammatory state. One scenario is that disruption of metabolic regulation in adipocytes results in increased efflux of free fatty acids (FFAs), which could activate locally resident macrophages, adding to the pro-inflammatory milieu in the AT. This would further enhance lipolysis, thereby establishing a self-reinforcing pro-inflammatory feedback loop (Suganami et al. 2005). EDCs could disrupt metabolic regulation in a number of ways, including *a*) nonspecific binding to multiple different nuclear receptors (NRs) (Bility et al. 2004); *b*) selective binding to pleiotropic NRs (Grün and Blumberg 2006); and *c*) epigenetic changes leading to an alteration of DNA methylation (Rajesh and Balasubramanian 2014). Due to their exogenous origin, EDCs cannot be readily placed into the context of a canonical biochemical or signaling pathway. In this light, a data-driven (e.g., multi-omic) approach could provide valuable clues in determining the pathways impacted by the chemical, which in turn could lead to mechanistic insights.

In this study, we combined metabolomic and proteomic analyses to study the biochemical changes elicited by a pervasive EDC, monoethylhexyl phthalate (MEHP), in differentiated adipocytes. Compared with two other representative EDCs, tributyltin (TBT) and bisphenol A (BPA), MEHP more drastically alters the cellular metabolic profile, while also eliciting a pro-inflammatory response in a dose-dependent fashion. Results of proteomic analysis in conjunction with gene expression data pointed to the involvement of the nuclear receptor peroxisome proliferator activated receptor-γ (PPARγ) as a mediator of the observed metabolic and inflammatory responses. Inhibition of PPARγ activity abrogated both the metabolic and inflammatory effects of MEHP, further supporting the involvement of PPARγ.

## Methods

### Chemicals and Reagents

Newborn calf serum (CS), fetal bovine serum (FBS), Dulbecco’s Modiﬁed Eagle’s Medium (DMEM), penicillin, streptomycin, insulin, phosphate-buffered saline (PBS), and TRIzol® reagent were purchased from Life Technologies, trypsin from Thermo Scientific, and recombinant mouse TNF-α from R&D Biosystems. Unless otherwise noted, all other chemicals and reagents were purchased from Sigma Aldrich.

### Cell Culture

Low passage 3T3-L1 preadipocytes (ATCC) were seeded into 12 well plates at a concentration of 10^5^ cells/cm^2^ and cultured in a humidified incubator at 37°C and 10% CO_2_. The cultures were expanded in a growth medium consisting of DMEM supplemented with 10% (vol/vol) CS, penicillin (100 units/mL), streptomycin (100 mg/mL), and amphotericin (2.5 mg/mL). The growth medium was changed every 2–3 days until the culture reached confluence. Two days postconfluence (Day 0), the cells were induced to differentiate using an adipogenic cocktail [1 mM dexamethasone, 1 mg/mL insulin, and 0.5 mM methylisobutylxanthine, (DIM)] added to a basal medium [DMEM with 10% (vol/vol) FBS and penicillin, streptomycin, and amphotericin]. After 48 hr, the DIM medium was aspirated, and the cells were fed fresh basal medium supplemented with only insulin. On Days 4 and 6, the cells were again fed the DIM and insulin medium, respectively, to complete the differentiation. On Day 8, the cultures were randomly divided into four treatment groups, and fed the basal medium supplemented with 100 nM TBT, BPA, MEHP, or 0.1% dimethyl sulfoxide (DMSO) (vehicle control). For the dose–response experiments, the treatment groups were fed the basal medium supplemented with 0.1, 1, or 10 μM MEHP. A fourth, control group was fed the basal medium supplemented with vehicle (0.01% DMSO) alone. For the inhibitor experiment, cells were fed basal medium supplemented with 10 μM MEHP or vehicle, in the presence or absence of 5 μM GW9662. The culture medium was replenished every other day for the remainder of the experiment. On Day 15, TNF-α (20 ng/mL) was added to a subset of the vehicle control cultures to generate a positive control for acute inflammation. Table 1 summarizes the timeline of the cell culture experiments. On Days 8, 12, and 16, images were recorded for a set of randomly selected wells from each treatment or control group, and then sacrificed for metabolomic and proteomic analysis. A second set of wells was sacrificed and the cells lysed using 0.1% sodium dodecyl sulfate (SDS) for biochemical assays of total triglyceride (TG), DNA, and protein content. A third set of wells was sacrificed for qPCR analysis. Sacrificing separate sets of wells in parallel was necessary due to the different lysis/extraction buffer requirements for mass spectrometry (MS), biochemical assays, and quantitative polymerase chain reaction (qPCR).

**Table 1:** Timeline of cell culture experiments.

Time (day)	–5	0	2	4	6	8^*a*^
Culture medium	Growth	DIM	Basal with insulin	DIM	Basal with insulin	Basal with EDC or vehicle
^***a***^TNF-α added on Day 15.						

### Metabolite Extraction

Metabolites were extracted from adherent cells using direct cell scraping followed by the application of a solvent mixture of methanol, chloroform, and water (Dettmer et al. 2011). After removing the culture medium and rinsing the cells with PBS, ice-cold methanol:water (91:9, vol/vol) was added (0.525 mL/well) to lyse the cells. Any remaining adherent cells and debris were scraped off the bottom to collect the entire contents of the well, which were transferred into a clean sample tube. After adding 0.475 mL of chloroform, the sample tube was vortexed vigorously to obtain a mono-phase mixture. The samples were subjected to 3 freeze-thaw cycles, and then centrifuged under refrigeration (4°C) at 15,000 × *g* for 5 min. The supernatant and pellet were separately collected for metabolite analysis and protein extraction, respectively. The supernatant samples were concentrated by evaporation in a speedvac concentrator, and then reconstituted in methanol:water (1:1, vol/vol). Extracted samples were stored at –80°C until analysis.

### Metabolomics

Targeted analysis of metabolites was performed using several different liquid chromatography–mass spectrometry (LC-MS) methods (see “LC-MS for metabolomics” in the Supplemental Material). For each LC-MS method, high-purity standards of the target metabolites were used to optimize compound-dependent parameters (e.g., collision energies) and identify product ions to monitor for quantification. For each detected target metabolite, the corresponding peak in the extracted ion chromatogram (XIC) was manually integrated using MultiQuant (version 2.1; AB Sciex) to determine the area under the curve (AUC). Absolute concentrations were determined from standard curves generated using the high-purity standards, and normalized to the corresponding sample DNA content.

### Protein Extraction

Cellular protein was extracted from the same cell lysate samples used for metabolite extraction. The method is based on a previously described protocol for plant cells (Weckwerth et al. 2004), which we modified for tissue culture samples by adjusting the solvent compositions and ratios. The extraction buffer was an aqueous solution of 0.05 M Tris (pH 7.6), 0.5% (weight/vol) SDS, and 1% (v/v) β-mercaptoethanol. Equal volumes (650 μL) of the extraction buffer and TRIzol^®^ reagent (Life Technologies) were mixed and added to the cell pellet collected from the metabolite extraction. After incubating for 1 hr at 37°C, the sample was vortexed and centrifuged under refrigeration (4°C) at 14,000 × *g* for 15 min to obtain phase separation. The upper and bottom phases containing RNA and protein, respectively, were separately collected into clean sample tubes using a syringe. The bottom phase was mixed with 1 mL ice-cold acetone, stored at –20°C overnight (18 hr), and centrifuged the next day under refrigeration (4°C) at 14,000 × *g* for 15 min to pellet the proteins. The protein pellet was washed three times with 1 mL ethanol. The precipitated protein was reduced, alkylated and digested into peptides using trypsin. Briefly, the proteins were reduced by incubating the sample at 37°C for 30 min with dithiothreitol (DTT) and 8 M urea. The next step added iodoacetamide and incubated the mixture for 15 min in the dark to alkylate cysteine residues. The digest step added trypsin to the reaction mixture at a ratio of 10 μg protease per 1 mg protein. After an overnight incubation, addition of formic acid terminated the reaction by lowering the pH to 2. Before the LC-MS analysis, a final centrifugation step removed any remaining undigested protein.

### Proteomics

We performed a series of untargeted experiments using the quadrupole-time of flight (QTOF) instrument to detect and quantify intracellular proteins. Chromatographic separation was achieved on an RP column (Ascentis® Express C18; Sigma Aldrich) using a gradient method involving two mobile phases (see “LC-MS for metabolomics” in the Supplemental Material). The MS experiments were information-dependent acquisition (IDA) and data-independent acquisition (DIA). An IDA scan was used to generate an ion library in ProteinPilot™(version 5.1; AB Sciex) of all proteins and their corresponding peptides in the sample, and a DIA (sequential window acquisition of all theoretical spectra, SWATH) scan was used to obtain high-quality MS/MS data for quantification (see “MEHP quantitation in media and cell extracts” and “LC-MS for proteomics” in the Supplemental Material). For each protein of interest identified from the IDA scan, the peptide(s) that gave the strongest signal intensity in the MS/MS spectra were manually selected to build a quantification method. A representative DIA scan data file was opened in PeakView® (version 1.2; AB Sciex) to verify the choice of product ions for quantification (see Figure S1). The peptides of interest were quantified using MultiQuant™ (version 2.1; AB Sciex) by first summing the intensities of the selected product ion peaks, and then integrating the summed intensities to determine the corresponding AUC. Changes in protein levels between different samples were calculated based on the AUC values normalized to the corresponding sample’s total ion current (TIC) from the time of flight (TOF)-MS survey scan, as well as to total protein content.

### Gene Expression Analysis

Cell samples were homogenized in TRIzol® reagent and total RNA was extracted according to the manufacturer’s instructions. RNA concentration and quality was assessed using a NanoDrop spectrophotometer (Thermo Scientific). Reverse transcription was performed using the Superscript® III First-Strand Synthesis System (Life Technologies), with 2 μg total RNA reverse transcribed using oligo(dT) primers. qPCR was performed with Brilliant II SYBR® Green qPCR Master Mix (Life Technologies) and the MX3000p qPCR System (Agilent). The primer pairs (Eurofins MWG Operon Oligos) used for qPCR analysis are listed in Table S1. Expression levels were calculated using the delta-delta cycle threshold method. Data are expressed as (log_2_) fold changes (normalized to 18S rRNA) relative to vehicle control.

### Biochemical Assays

Assays of total DNA, protein, and cellular TG content used cell samples lysed and sonicated in the SDS buffer. Total DNA was measured with a fluorescence-based assay using the Hoechst dye method. Total protein content in a sample was determined using a BCA assay kit (Thermo Scientific) per the manufacturer’s instructions. Cellular TG content was measured using an enzymatic assay kit (Sigma Aldrich) as described previously (Si et al. 2009).

### Statistical Analysis

We used partial least squares discriminant analysis (PLS-DA) to compare the metabolite profiles of the treatment groups. Prior to the analysis, the metabolite data were standardized to unit variance and zero mean. Calculations of latent variable (LV) scores and loadings were performed in MATLAB (version R2015b; Mathworks). The first two LV scores and corresponding loadings were plotted for each sample to visualize sample groupings and identify discriminatory metabolites. For each treatment group, a confidence ellipse was drawn to define the region that contains 95% of all samples that can be expected from the underlying Gaussian distribution. Mahalanobis distance was used to determine the separation between group centroids, and the corresponding *p*-values are reported in the figure legends for treatment groups showing significant separation from control.

Pairwise comparisons were performed using the Student’s *t*-test (version Office 2011; Microsoft Excel). The threshold for significance was set at *p* < 0.05. For qPCR data, error bars were drawn using a weighted standard deviation (s) of the error in the control and treatment group delta Ct values, where s = [(stdev(treatment ΔCt))^2^ + (stdev(control ΔCt))^2^]^1/2^. For repeat experiments, the standard deviation was calculated based on the average fold-change for all biological replicates.

## Results

### EDC Exposure Increases Expression of Inflammatory Gene Markers

**Figure 1:**
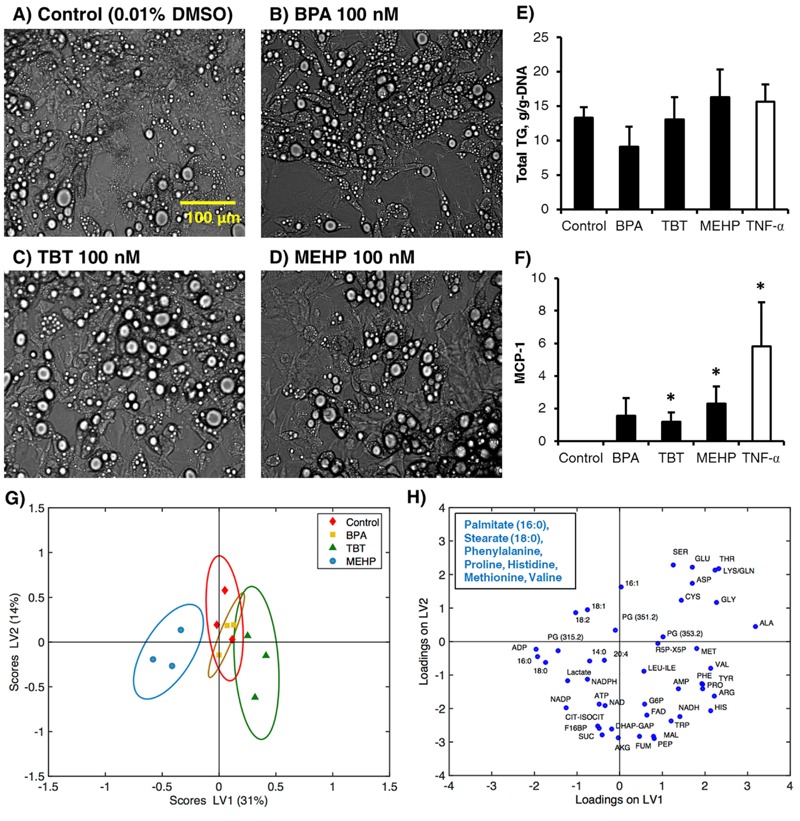
Effects of EDCs on lipid accumulation, inflammation, and metabolite profiles in differentiated adipocytes. (*A*–*D*) Representative bright-field images of cells recorded 8 days after EDC treatment (Day 16). Scale bar: 100 μm. (*E*) Total cellular triglyceride in each culture well normalized to the corresponding DNA content. (*F*) MCP-1 gene expression on Day 16. (*G*) PLS-DA scores of Day 16 metabolite data for different EDC treatments with 95% confidence ellipses. Each point represents the average of 3 biological replicates from 1 experiment. (*H*) Discriminatory metabolites identified based on the loadings are listed in the boxed insert. Note: All data shown represent the number (N = 3) of independent repeat experiments. Each independent experiment had *n *= 3 samples, which we refer to as biological replicates (3 biological replicates per experiment). Error bars show standard deviation (SD). *p-Value < 0.05.

Based on published reports suggesting that TBT, BPA, and MEHP are obesogens capable of stimulating lipogenesis (Feige et al. 2007; Grün et al. 2006; Sargis et al. 2010), we first characterized the effects of these chemicals on lipid accumulation in differentiated 3T3-L1 adipocytes. Compared with previous studies (Chiang et al. 2016; Ellero-Simatos et al. 2011), we applied substantially lower doses that are comparable to systemic concentrations found in human exposure studies (Frederiksen et al. 2010; Kannan et al. 1999; Vandenberg et al. 2012). For the duration of the exposure experiment (Days 8–16), the cells remained rounded and lipid laden, and no qualitative differences in cellular and LD morphology were observed between the treatment groups (Figure 1A–D). Quantitative assessment of total cellular triglyceride content did not show any significant differences between the treatment groups (Figure 1E). Gene expression analysis showed that both TBT and MEHP significantly increased the transcript level of monocyte chemoattractant protein-1 (MCP-1), a major chemotactic factor that signals for recruitment and M1 polarization of macrophages (Figure 1F).

### Metabolomics Indicate MEHP Is a Potent Metabolic Disruptor

To broadly assess the metabolic effects of EDC exposure, the levels of 46 central carbon metabolites were measured using LC-MS. PLS-DA was performed to determine whether there were significant differences in the metabolite profiles between the treatment groups, and if so, to identify which metabolites contributed to discriminating the groups from each other. Projections of the metabolite profiles onto the first and second LV space showed a clear separation between the MEHP treatment and control groups along the first LV (Figure 1G). The MEHP group projected furthest from the control group, with a Mahalanobis distance of 6.89 compared with a distance of 1.86 and 2.35 for TBT and BPA, respectively. Statistical testing (χ^2^ distribution; Mathworks) at the 95% significance level confirmed that the separation between the MEHP and control groups is significant.

### Multivariate Analysis Reveals Discriminatory Metabolites

The loadings plot from PLS-DA represents the influence of the metabolites on the separation between treatment groups, with each loading corresponding to a distinct metabolite. Of the five metabolites that were uniquely different for MEHP relative to control (Figure 1H, box insert), the two metabolites positively correlated with MEHP exposure are fatty acids, suggesting that MEHP exposure led to major changes in lipid metabolism. Other lipids contributing to the separation of MEHP and control include both saturated and unsaturated long-chain fatty acids as well as complex lipid derivatives (designated as ‘PG’ in Figure 1H) that are likely prostaglandins.

### MEHP Upregulates Pro-inflammatory Cytokine and Chemokine Expression

**Figure 2:**
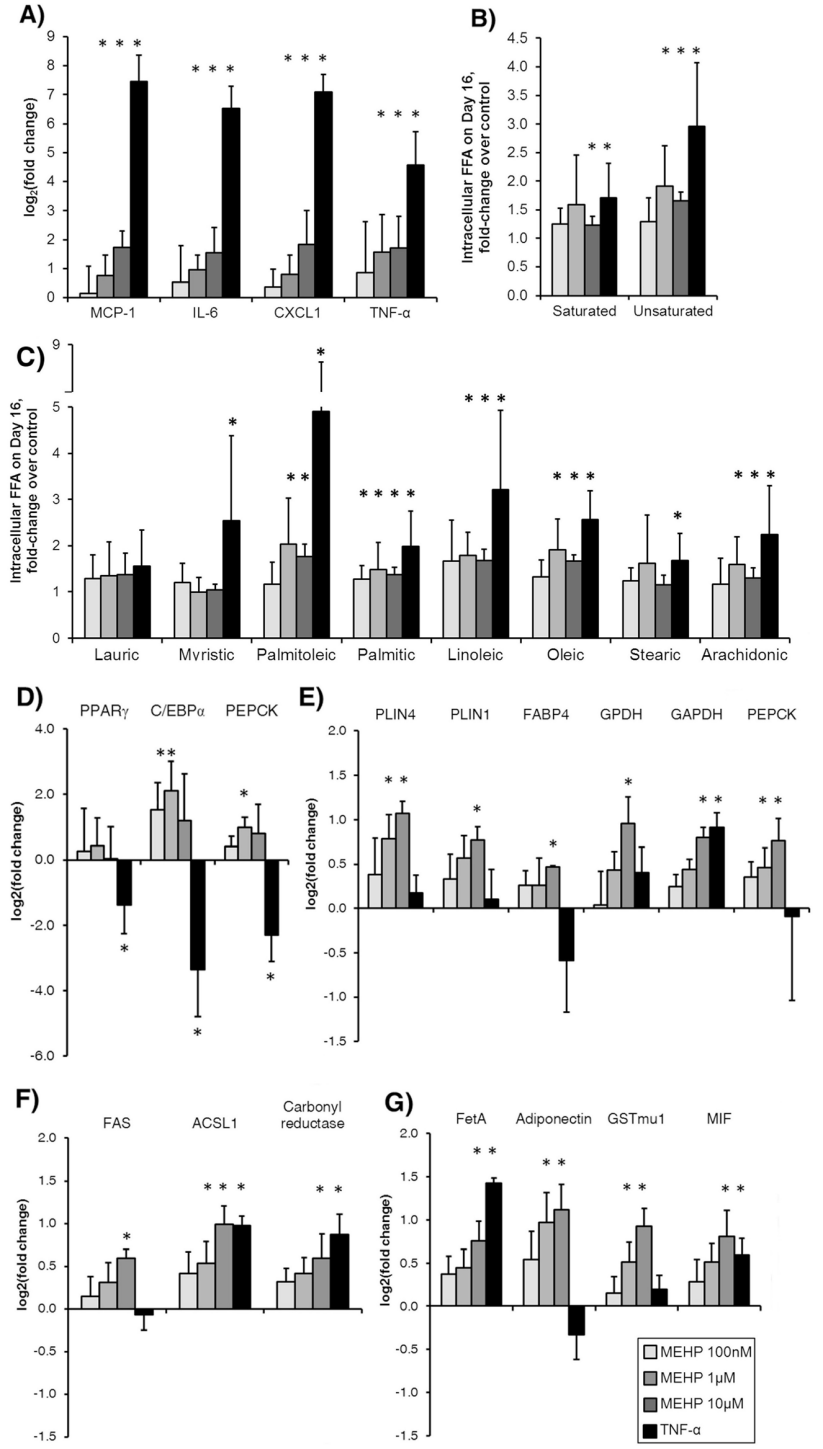
Dose effects of MEHP on adipocyte inflammatory markers, fatty acid profile, and proteins mediating inflammatory signaling and regulating lipid metabolism. (*A*) Expression profiles of inflammatory gene markers. (*B*) Summed contributions of quantitatively significant unsaturated and saturated FFAs. (*C*) Intracellular FFA profile. Data shown are the average of N = 2, which refers to independent repeat experiments. Each independent experiment in this case had *n *= 3 samples, which we refer to as biological replicates (3 biological replicates per experiment). (*D*) Gene expression profiles of PPARγ, its cross activator C/EBPα, and an enzyme target. Data shown are averages of *n* = 3 biological replicates. (*E*) Relative abundance of PPARγ targets, (*F*) lipid metabolizing enzymes, and (*G*) inflammatory mediators. Values are (log_2_) fold changes in protein abundance relative to vehicle control. Data shown are averages of *n* = 4 biological replicates. Protein abundance quantified from sequential window acquisition of all theoretical spectra (SWATH) MS data was normalized to total protein and total ion current (TIC). Error bars show SD. *p-Value < 0.05.

We next performed a dose response experiment to characterize in more detail the chemical’s effects on inflammation indicators and metabolic pathways. We first confirmed that MEHP was taken up from the culture medium, and that this uptake occurred in proportion to the administered dose (see Figure S2). Gene expression analysis showed significant upregulation of pro-inflammatory cytokines (TNF-α and IL-6) and chemokines (MCP-1 and CXCL1) in MEHP-treated cells on Day 16, albeit to a lesser extent than the positive control TNF-α (Figure 2A).

### MEHP Raises Intracellular Free Fatty Acid Levels

To quantitatively characterize the MEHP-induced changes in metabolism, absolute concentrations were determined for central carbon metabolites using a targeted LC-MS analysis. We first examined the most discriminatory metabolites identified from the PLS-DA. MEHP treatment significantly altered the FFA profile on Day 16, significantly raising the levels of both saturated and unsaturated fatty acids (Figure 2B). Examining specific FFAs (Figure 2C), we found that exposure to MEHP (1–10 μM) significantly increased the intracellular levels of palmitoleic, palmitic, linoleic, oleic, and arachidonic acid. Treatment with TNF-α, which is known to stimulate lipolysis in adipocytes, also increased the levels of these FFAs. In addition to fatty acids, MEHP broadly altered the levels of other central carbon metabolites (see Figure S3), including glycolysis and TCA cycle intermediates.

### MEHP Alters PPAR *γ* Target Enzyme Expression and Abundance

Based on the observation that MEHP treatment significantly perturbed the profile of intracellular fatty acids, we investigated the chemical’s effects on key regulators of lipid metabolism. Gene expression data showed that MEHP exposure did not alter the mRNA level of PPARγ. However, the expression levels of two PPARγ targets, CCAAT/enhancer-binding protein alpha (C/EBPα) and phosphoenolpyruvate carboxykinase (PEPCK), were increased significantly (Figure 2D), implying increased PPARγ activity.

The responses of PPARγ and two of its target genes suggested that gene expression analysis alone does not fully capture the impact of MEHP exposure on PPARγ-regulated pathways. Therefore, we combined untargeted proteomics with targeted data extraction to profile the levels of cellular proteins. Overall, the proteomic data revealed significant changes in the levels of metabolic enzymes and other proteins involved in regulating the lipid balance in adipocytes (see Figure S4). Significantly upregulated proteins include glycerol-3-phosphate dehydrogenase (GPDH), PEPCK, perilipins 1 and 4 (PLIN1/4), fatty acid binding protein 4 (FABP4), and adiponectin, all downstream targets of PPARγ (Figure 2E). In addition to these PPARγ target proteins, MEHP exposure increased the levels of several other enzymes involved in lipid metabolism, including acyl-CoA synthetase long-chain family member 1 (ACSL1), fatty acid synthase (FAS), and carbonyl reductase (Figure 2F).

The proteomic analysis also revealed alterations in the levels of inflammatory signaling proteins. Exposure to MEHP at the highest dose (10 μM) led to a 2-fold increase in the pro-inflammatory cytokine macrophage migration inhibitory factor (MIF), which was also increased upon TNF-α treatment (Figure 2G). In addition, we observed a significant increase in the abundance of fetuin A (FetA), a peptide factor that stimulates macrophage migration and polarization in AT. Interestingly, we found signs of stress response to xenobiotic accumulation, specifically an increase in the glutathione S-transferase mu 1 (GST mu1). This increase was not observed in the TNF-α condition, suggesting that the response is specific to xenobiotic chemicals, rather than inflammation.

### PPAR *γ* Inhibition Attenuates MEHP-Induced Gene and Protein Changes

**Figure 3:**
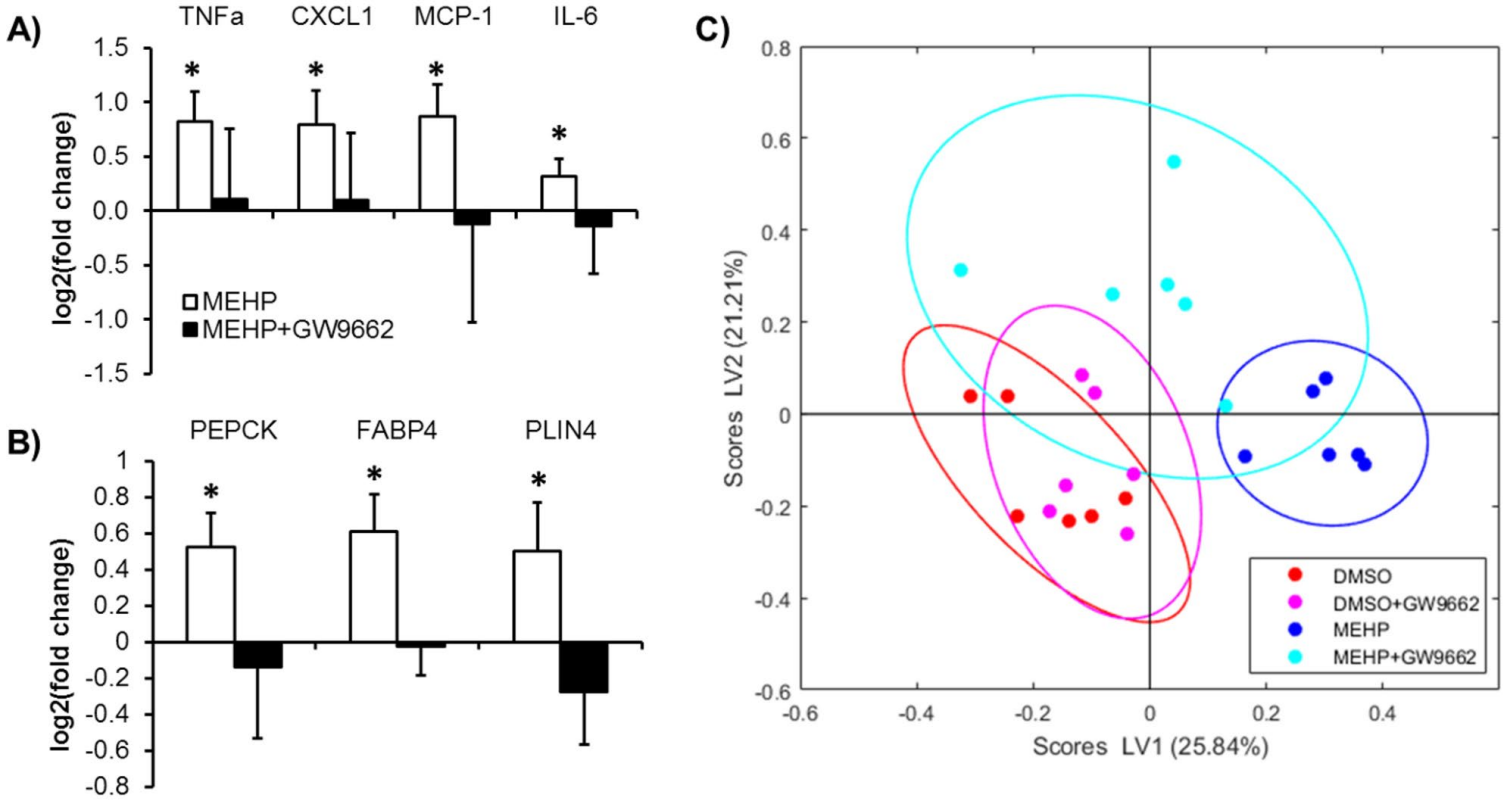
Adipocytes co-treated with MEHP and GW9662 are more phenotypically similar to vehicle controls than cells treated with MEHP alone. (*A*) Expression profiles of inflammatory gene markers. (*B*) Relative abundance of PPARγ targets. Values are (log_2_) fold changes in protein abundance relative to vehicle control. Data shown are averages of N = 3 independent experiments (3 biological replicates per experiment). (*C*) Scatter plot of PLS-DA scores for untargeted proteomics data showing significant separation between vehicle control and MEHP groups. For 10 μM MEHP, *p* = 0.03. Note: Data shown represent the number (N = 2) of independent repeat experiments. Each independent experiment in this case had *n *= 3 samples, which we refer to as biological replicates (3 biological replicates per experiment). Error bars show standard deviation. *p-Value < 0.05.

To investigate the involvement of PPARγ in the responses elicited by MEHP, we repeated the exposure experiments in the presence of the selective PPARγ antagonist GW9662. We observed significant attenuation of the MEHP-induced expression of pro-inflammatory cytokines and chemokines (Figure 3A), which suggested that PPARγ could recognize MEHP as an activator. GW9662 also attenuated levels of PPARγ target proteins upregulated by MEHP (Figure 3B).

A broader comparison of protein expression profiles revealed a similar trend. PLS-DA on untargeted proteomic data showed that treatment with MEHP significantly altered the intracellular protein profile of mature adipocytes by Day 16 (Figure 3C). These alterations were significantly attenuated by co-treatment with GW9662. In comparison, the protein profile of cells in the vehicle control group was not significantly impacted by treatment with GW9662.

## Discussion

This study demonstrates for the first time that MEHP directly stimulates inflammation in differentiated adipocytes. Importantly, the doses employed in this study reflect physiologically relevant concentrations detected in human populations (Frederiksen et al. 2010; Kannan et al. 1999; Vandenberg et al. 2012). We combined metabolomics with gene expression analysis to compare the effects of three representative EDCs on adipocyte metabolism and inflammation. Of the three EDCs tested, MEHP drastically altered the metabolic profile of adipocytes, while also upregulating inflammation markers. Thus, this EDC was selected for further investigation to characterize in more detail the pathways involved in mediating these effects.

A majority of previous studies aimed at monitoring EDCs in humans have focused on blood or urine samples (Carwile and Michels 2011; Genuis et al. 2012), with only a handful of studies analyzing local EDC levels in the AT. *In vitro* studies offer the opportunity to characterize the responses of a specific cell type to a chemically defined EDC in isolation from systemic influences. Using a quantitative LC-MS assay, we confirmed that MEHP depletes from the culture medium and accumulates inside the cells (see Figure S2). A material balance indicated that MEHP, once taken up by the cells, is only partially transformed (67%, 66%, and 25% for the 10, 1, and 0.1 μM dose, respectively).

Previous studies on the effects of MEHP in adipocytes have focused mostly on differentiation, and generally report a pro-adipogenic effect. Experiments in 3T3-L1 preadipocytes showed that MEHP stimulates differentiation in the presence of insulin (Siriwardhana et al. 2012). Similar trends were observed in primary human adipocytes derived by *in vitro* differentiation of freshly isolated preadipocytes (Ellero-Simatos et al. 2011); however, a relatively high dose of 100 μM was required to produce significant effects. Unlike these earlier studies, we did not observe any obvious adipogenic effects such as increases in total triglycerides. The main effects were upregulation of inflammation related genes and proteins along with broad changes in the levels of metabolites and enzymes.

To our knowledge, there has not been any prior evidence for phthalate-induced inflammation in adipocytes. A recent study reported signs of systemic and local inflammation in rats exposed to DEHP *in utero* (Campioli et al. 2014). Abdominal fat pads from these animals showed increased TNF-α expression in the stromal vascular fraction, pointing to the resident macrophages and monocytes as the source of the cytokine. The results of our study show that MEHP elicits an inflammatory response in differentiated adipocytes independent of immune cells. This clearly does not rule out the possibility that the *in vivo* response involves both adipocyte and immune cell contributions. Rather, the *in vivo* response likely involves cytokine-mediated paracrine signaling between the different cell types. Indeed, we found that MEHP exposure significantly increased gene expression of TNF-α, IL-6, MCP-1, and CXCL1, which are characteristically elevated in inflamed AT (Makki et al. 2013; Nunemaker et al. 2014).

To examine whether MEHP exposure affected inflammatory signaling at the protein level, while also obtaining a more complete picture of the chemical’s effects, we conducted an untargeted proteomic analysis. Using PLS-DA, we identified 50 highly significant discriminatory proteins based on their loading values (see Table S2). A majority (58%) of these proteins has known functions in inflammation, metabolism, or stress response (see Figure S4). Several of the discriminatory proteins mediate monocyte recruitment and homeostasis. Therefore, we quantitatively analyzed additional proteins related to adipocyte-monocyte interactions. We observed a significant increase in FetA, which promotes macrophage infiltration and polarization in AT. Release of FetA from cultured adipocytes is stimulated by FFAs (Chatterjee et al. 2013), suggesting a link to lipid metabolism. Another intriguing trend is the dose-dependent increase in MIF, which acts as a pro-inflammatory signal in AT and positively associates with obesity-related insulin resistance (Finucane et al. 2012). Studies in mice and cultured adipocytes suggest that MIF exerts catabolic effects by inhibiting insulin signaling (Atsumi et al. 2007), pointing to another potential link between the metabolic and inflammatory effects of MEHP exposure.

This connection is further supported by the results of our metabolomic analysis. The LV loadings from PLS-DA identify fatty acids as key discriminatory metabolites (see Figure S3). These include arachidonic acid, which is a major source of eicosanoids that mediate inflammatory signaling. In cultured adipocytes, arachidonic acid stimulates secretion of MCP-1 and IL-6 in a dose-dependent fashion (Siriwardhana et al. 2012). Quantification of abundant fatty acids showed a general pattern of increase with MEHP treatment compared to control samples, resembling the intracellular profile in TNF-α treated cells (Figure 2B,C). Whether this increase results from enhanced lipolysis as in the case of TNF-α (Zhang et al. 2002) is unclear.

Over-representation analysis (Ingenuity Pathway Analysis; Qiagen) of the discriminatory proteins from PLS-DA as well as metabolite profiles also pointed to the activation of inflammatory pathways in response to MEHP exposure. Four of the top five significant (*p* < 0.05) diseases or biofunctions associated with the omic profiles were related to inflammation and immune response. The most significantly affected metabolic function is lipid metabolism, underscoring a possible link between the altered fatty acid levels and inflammatory phenotype.

Consistent with previous studies showing that MEHP induces transcription of PPARγ targets (Ellero-Simatos et al. 2011; Feige et al. 2007; Siriwardhana et al. 2012), our results suggest that MEHP could interfere with PPARγ signaling to disrupt regulation of cellular lipid metabolism (Figure 2D). In addition to upregulation of C/EBPα, we found increases in several enzymes and lipid regulatory proteins (FABP4, PLIN4, PEPCK, GPDH, and adiponectin) that are known PPARγ targets. Normally, upregulation of anabolic enzymes occurs in parallel with counter-regulation of catabolic enzymes, promoting fatty acid esterification. However, this was not the case in MEHP exposed cells, suggesting at least partial dysregulation. We observed a significant upregulation of ACSL1, which activates the breakdown of complex fatty acids, and thus impacts the profile of endogenous ligands for PPARγ. To further investigate the involvement of PPARγ in the observed metabolic and inflammatory effects, we tested whether inhibiting PPARγ activity would abrogate the effects of MEHP exposure. A comparison of protein expression profiles showed that co-treating the cells with the selective synthetic antagonist GW9662 during MEHP exposure attenuated MEHP-induced differences, taking the adipocytes to a phenotypic state more similar to vehicle control. Furthermore, co-treatment with MEHP and GW9662 prevented the significant increase in chemokine and cytokine expression observed when the cells were treated with MEHP alone. To further boost confidence in our finding that PPARγ plays a role in mediating the effects of MEHP, we performed a pathway enrichment analysis using the STRING database (Jensen et al. 2009). The analysis found a total of 14 significant pathways (Table 2). Of these, 6 pathways represented either generic or disease specific function categories. Of the remaining 8 pathways, 6 were metabolic pathways, and the other two were PPAR signaling and actin cytoskeleton regulation. Based on the *p*-value, the top two pathways were fatty acid degradation and metabolism. Taken together, these results suggest that PPARγ activation is at least partially responsible for mediating the metabolic and inflammatory effects induced by MEHP treatment.

**Table 2:** Significantly enriched KEGG pathways in MEHP condition.

KEGG pathway	Number of genes	*p*-Value^*b*^
Carbon metabolism^*a*^	8	2.46E-07
Microbial metabolism in diverse environments^*a*^	8	9.13E-06
Systemic lupus erythematosus^*a*^	6	7.53E-05
Metabolic pathways^*a*^	16	8.06E-05
Alcoholism^*a*^	6	9.48E-04
Fatty acid degradation	4	2.53E-03
Fatty acid metabolism	4	3.88E-03
Valine, leucine and isoleucine degradation	4	3.88E-03
Glycolysis/Gluconeogenesis	4	9.14E-03
Regulation of actin cytoskeleton	6	1.03E-02
Arrhythmogenic right ventricular cardiomyopathy^*a*^	4	1.84E-02
Biosynthesis of amino acids	4	1.84E-02
PPAR signaling pathway	4	2.26E-02
Propanoate metabolism	3	2.84E-02
Note: KEGG, Kyoto Encyclopedia of Genes and Genomes. ^***a***^Generic or disease-specific function categories. ^***b***^*p*-Values were corrected for multiple comparisons using the Bonferroni method.		

One regulatory mechanism that could be disrupted by MEHP is feedback between PPARγ and FABP4. As a carrier protein that transports long-chain FFAs to their cognate receptors in the nucleus, FABP4 is a key link in FFA-mediated NR signaling. FABP4 can attenuate PPARγ activity by triggering its proteasomal degradation, and FABP4-null mice exhibit increased expression of PPARγ (Garin-Shkolnik et al. 2014). Another study found that treatment of adipocytes with FFA downregulated PPARγ protein and mRNA levels while upregulating inflammatory indicators (Nguyen et al. 2012), suggesting that maintaining a certain level of PPARγ activity is important in modulating inflammation. In the context of MEHP exposure, sustained exogenous stimulation of PPARγ by the chemical could result in loss of activity via inhibitory factors such as FABP4, impairing the NR’s ability to regulate against buildup of FFA. Increased levels of FABP4 have been observed in AT of obese-diabetic individuals (Garin-Shkolnik et al. 2014), which characteristically exhibit a low-grade inflammation.

## Conclusions

We found a strong correlation between MEHP-induced changes in lipid metabolism and upregulation of pro-inflammatory cytokines at gene expression, protein, and metabolite levels, suggesting these responses are coupled. Results from the PPARγ inhibition experiment show that the NR is at least partially involved in mediating both the metabolic and inflammatory effects of MEHP. As a synthetic chemical, MEHP is not easily placed into the context of canonical signaling and metabolic pathways. Thus, we used a data-driven approach combining gene expression analysis, metabolomics, and proteomics to broadly characterize the biochemical changes induced by the chemical and to identify potential sites of action. Although additional studies are clearly necessary to establish whether the metabolic changes precede and cause inflammation, our results firmly indicate that MEHP exposure is sufficient to upregulate the expression of key pro-inflammatory factors in differentiated adipocytes independent of signals from other cell types. *In vivo,* autocrine and paracrine events between adipocytes and inflammatory cells would add to the cytokine milieu, sustaining a pro-inflammatory feedback loop in the AT (Suganami et al. 2005). In this way, prolonged exposure to MEHP could set the stage for chronic inflammation. Testing this potential outcome warrants further study, and will likely require long-term exposure experiments using low doses, preferably *in vivo*.

## Supplemental Material

(1.2 MB) PDF
