# Supplementary material for: Monoethylhexyl Phthalate Elicits an Inflammatory Response in Adipocytes Characterized by Alterations in Lipid and Cytokine Pathways
Source: Environ Health Perspect. 2016 Jul 6;125(4):615–22. doi: 10.1289/EHP464 (PMC5381996; doi:10.1289/EHP464)
Supplement: (1.2 MB) PDF [file EHP464.s001.acco.pdf]

Environ Health Perspect
DOI: 10.1289/EHP464

Note to readers with disabilities: *EHP* strives to ensure that all journal content is accessible to all readers. However, some figures and Supplemental Material published in *EHP* articles may not conform to 508 standards due to the complexity of the information being presented. If you need assistance accessing journal content, please contact ehp508@niehs.nih.gov. Our staff will work with you to assess and meet your accessibility needs within 3 working days.

Supplemental Material

Monoethylhexyl Phthalate Elicits an Inflammatory Response in Adipocytes Characterized by Alterations in Lipid and Cytokine Pathways

Sara Manteiga and Kyongbum Lee

Table of Contents

Supplemental Methods
- LC-MS for metabolomics
- MEHP quantitation in media and cell extracts
- LC-MS for proteomics

Supplemental Figures
- Figure S1. Illustration of SWATH data analysis.
- Figure S2. MEHP accumulates in adipocytes
- Figure S3. MEHP broadly alters metabolite levels in a dose dependent fashion.
- Figure S4. PLS-DA of untargeted proteomics

Supplemental Tables
- Table S1. Primer sequence used for qPCR
- Table S2. Discriminatory proteins from PLS-DA.

Supplemental Methods

LC-MS for metabolomics. Targeted analysis of metabolites was performed using LC-MS experiments on a quadrupole time-of-flight (QTOF 5600+, AB Sciex) and quadrupole ion-trap (QTRAP 3200, AB Sciex) instrument. The QTRAP was used for multiple reaction monitoring (MRM) scans performed in positive ion mode to analyze amino acids. Chromatographic separation was achieved on a C18 reverse phase (RP) column (Synergi Fusion-RP 4μ 80Å 150 x 2.0 mm, Phenomenex) using a gradient method. Solvent A was water + 0.1% formic acid and solvent B was methanol + 0.1% formic acid. The flow rate was held constant at 100 μL/min. The mobile phase gradient was as follows. Solvent A was held at 97% from 0 to 8 min, linearly decreased to 5% over 30 min, held at 5% for 7 min, linearly increased to 97% over 2 min, and held at 97% for 8 min.

The QTOF was used to analyze fatty acids, nucleotide cofactors, and intermediates of glycolysis, TCA cycle, and pentose phosphate pathway (PPP). Fatty acids were analyzed using RP chromatography (Luna C8(2) 5μ 100 Å 150 x 2.0 mm, Phenomenex) and a TOF-MS scan in negative mode. Solvent A was a 3% (v/v) methanol solution in water + 10 mM tributylamine and 15 mM acetic acid (pH 4.5). Solvent B was methanol. The flow rate was held constant at 300 μL/min. The mobile phase gradient was as follows. Solvent A was initially set at 20%, linearly decreased to 1% over 20 min, held at 1% for 20 min, linearly increased to 20% over 2 min, and held at 20% for 9 min. Glycolysis, TCA cycle, and PPP intermediates, and nucleotide cofactors were analyzed using hydrophilic interaction chromatography (HILIC) on an amide column (Luna NH_2 5μ 100Å 250 x 2.0 mm, Phenomenex) and a TOF-MS scan combined with product ion scans conducted in negative ion mode. Solvent A was a 5% acetonitrile solution in water (v/v) + 20 mM ammonium hydroxide and 20 mM ammonium acetate (pH 9.45). Solvent B was

acetonitrile. The flow rate was held constant at 200 µL/min. The mobile phase gradient was as follows. Solvent A was initially set at 15%, linearly increased to 100% over 5 min, held at 100% for 23 min, linearly decreased to 15% over 4 min, and held at 15% for 8 min.

MEHP quantitation in media and cell extracts. The amount of residual MEHP in the culture medium was quantified using a targeted LC-MS method performed on the QTRAP. The same LC method used to measure free fatty acids was paired with an MRM scan in negative mode set to record the transition *m/z* 277.1→133.9. Uptake by the cells was calculated based on the concentration difference in spent and fresh medium. Intracellular accumulation was determined based on the amount of MEHP in the cell extract.

LC-MS for proteomics. Chromatographic separation was achieved on a RP column (Ascentis Express C18 2.7 µm 100Å 150 x 2.1 mm, Sigma Aldrich) using a gradient method. Solvent A was water + 0.1% formic acid and solvent B was acetonitrile + 0.1% formic acid. The flow rate was held constant at 200 µL/min. Solvent A was held at 98% from 0 to 15 min, linearly decreased to 55% over 35 min, held at 55% for 10 min, linearly decreased to 5% over 2 min, held at 5% for 13 min, linearly increased to 98% over 0.5 min, and held at 98% for 4.5 min.

The IDA method comprised a TOF MS (survey) scan and (triggered) high-resolution MS/MS (product ion) scans monitoring up to 25 candidate ions per cycle. The dependent scans were triggered whenever the survey scan detected a precursor ion matching the following criteria: mass range of *m/z* 300-1250, charge state of +2 to +5, mass tolerance of 50 mDa, exclude isotopes within 4 Da, ion count exceeds 100 cps. Previously fragmented target ions were excluded from fragmentation for 15 sec to improve the number of different ions fragmented.

Rolling collision energy ensured that fragment ions were generated for a wide variety of peptides. Proteins were identified using ProteinPilot (v. 5.1, AB Sciex) and the SwissProt database. For the DIA (SWATH) experiments, the mass spectrometer stepped through a set of 25 Da precursor acquisition windows covering a 400-1600 *m/z* mass range within a 3.33 sec cycle. During each cycle, the mass spectrometer fragmented all precursor ions in the quadrupole isolation window, and recorded a complete fragment ion spectrum for each precursor selected in that window. The same precursor isolation window was fragmented repeatedly at each cycle during the entire LC separation.

LC-MS data from the TOF-MS survey scan was processed in MarkerView (v. 1.2, AB Sciex) to identify and quantify peaks of ions with known *m/z* and retention time (RT). Peaks were aligned across samples by treating peaks from two samples with the same *m/z* (30 ppm tolerance) and RT (1 min tolerance) as the same variable. The number of aligned peaks used for further analysis was cut off at 10,000 based on intensity. The response (integrated peak area) for each variable (precursor ion) was then normalized by total peak area to account for variability in total protein loaded onto the column. PLS-DA was performed on the resulting data table in MATLAB (MathWorks). The loadings for the first 3 latent variables (LVs) were sorted by magnitude, and thresholds (97.5th percentile for LV1, and 99th percentile for LVs 2 and 3) were applied to select discriminatory peptide ions. Each discriminatory ion was assigned a peptide identity by manually searching the ion library generated in ProteinPilot (v. 5.1, AB Sciex) and its corresponding protein identity was determined directly through the ion library or by performing a BLAST search on the peptide sequence. Each identified protein was then categorized into one or more biological functions (inflammation, metabolism, stress response, and other) by searching the UniProtKB/Swiss-Prot database for related GO annotations.

Supplemental Figures

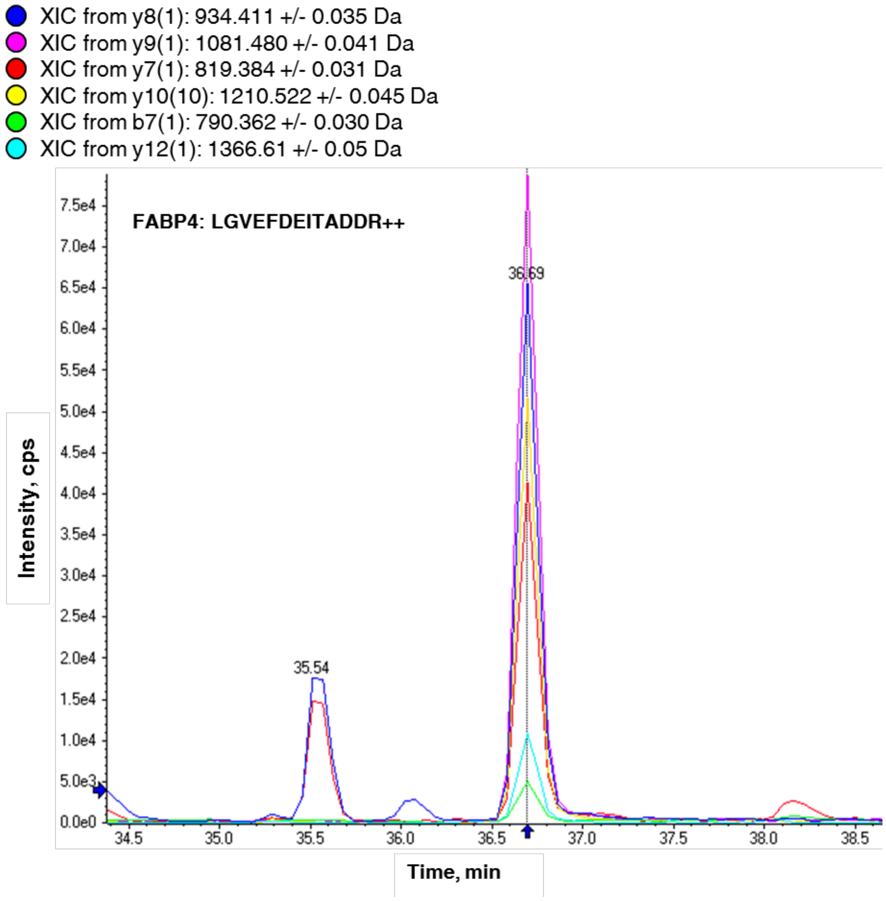

Figure S1. Illustration of SWATH data analysis.
Example shows MS/MS XIC of a peptide and its fragment ions used to identify and quantify fatty acid binding protein 4 (FABP4). Using PeakView, the various product ion *m/z* traces were extracted as XICs from the TIC of the isolation window that contains the precursor ion *m/z* value. The most intense product ions were selected for quantification.

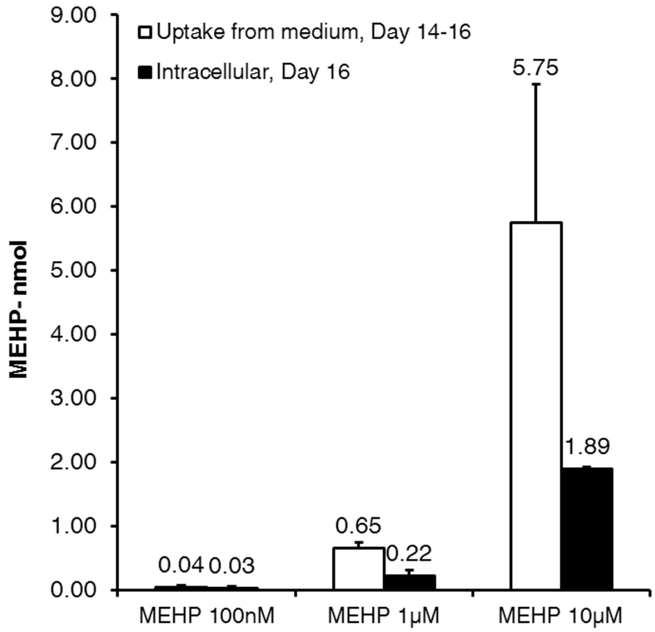

Figure S2. MEHP accumulates in adipocytes.
White bar: Amount of MEHP taken up from the culture medium between Days 14 and 16. Black bar: Amount of intracellular MEHP measured on Day 16. MEHP was last added to the culture on Day 14. Data shown are averages of N = 3 biological replicates.

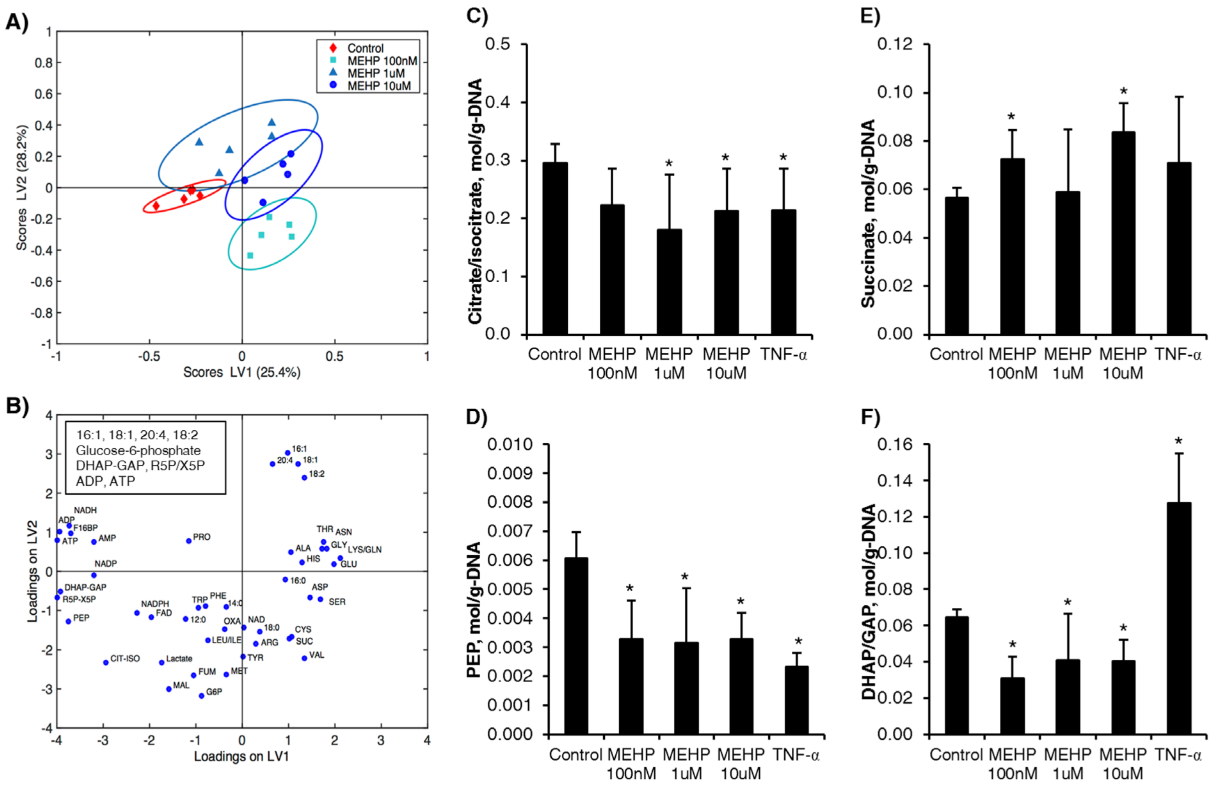

Figure S3. MEHP broadly alters metabolite levels in a dose dependent fashion.
(A) Ellipses around PLS-DA scores show regions of 95 % confidence. For 100nM and 10μM MEHP, p=0.047 and 0.01, respectively.(B) Discriminatory metabolites identified based on the loadings are listed in the boxed insert. (C-E) Intracellular levels of TCA and glycolysis intermediates on Day 16. Concentrations of the isomers citrate/isocitrate and DHAP/GAP are showed as lumped quantities. All data shown are averages of N = 5 biological replicates. (*P-value < 0.05). DHAP: dihydroxyacetone phosphate; GAP: glyceraldehyde 3-phosphate.

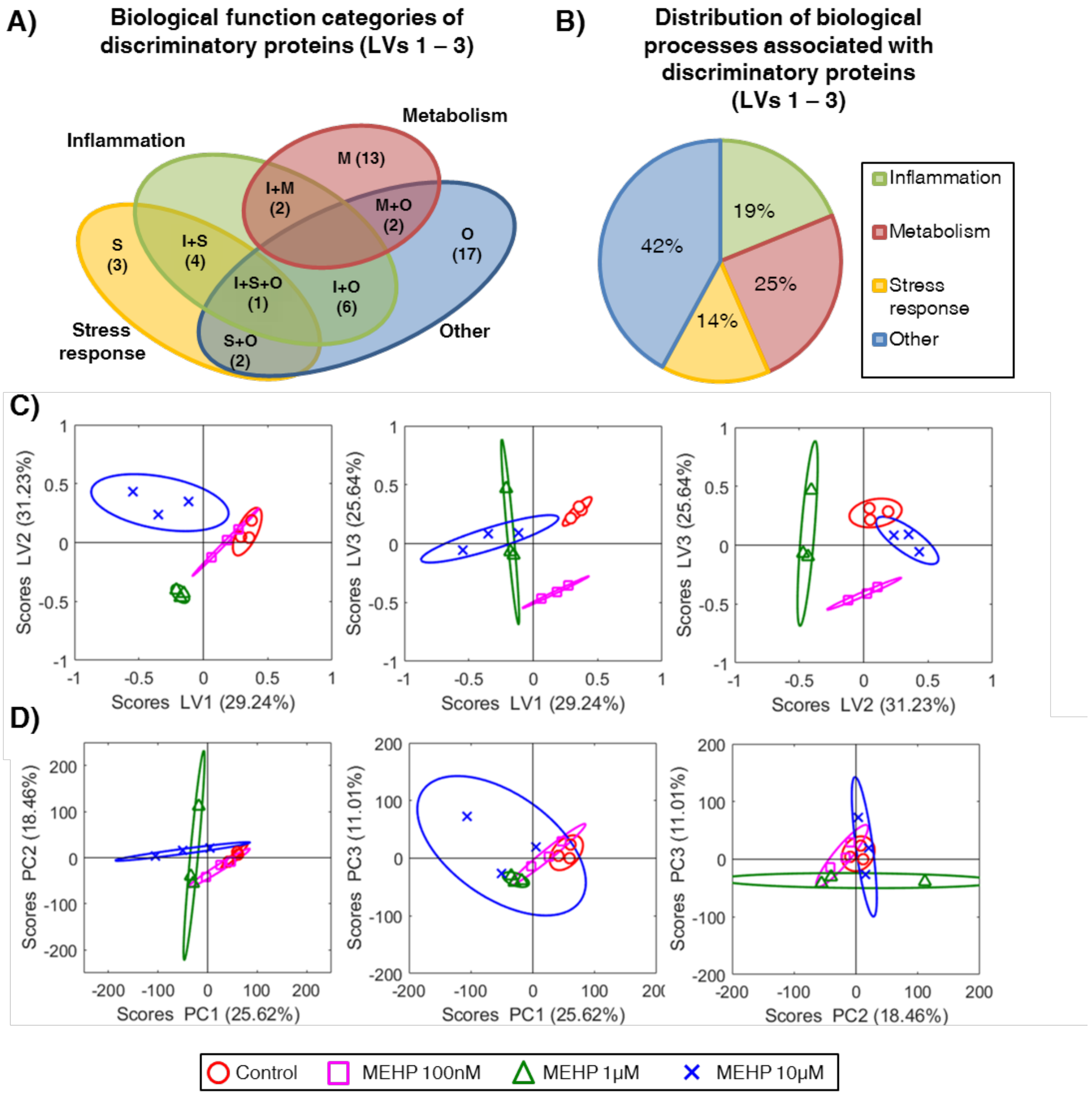

Figure S4. PLS-DA of untargeted proteomic data reveals a broad set of MEHP-induced changes in the levels of proteins that are involved in inflammatory signaling, metabolism, and stress response. (A) Discriminatory proteins identified based on the PLS-DA loadings were assigned one or more Gene Ontology (GO) categories of processes and/or functions. The number of proteins in the respective categories are shown in parentheses. I: inflammation; M: metabolism; S: stress response; O: other. Plus (+) symbol indicates multiple categories. (B) Distribution of processes associated with the discriminatory proteins for the first 3 latent variables (LVs). (C) Scatter plots of PLS-DA scores showing separation between treatment groups. For LV1&2 plot, p=0.01 for 10μM MEHP; For LV1&3 plot, p=0.0033 and 0.02 for 10 and 1μM MEHP, respectively; For LV2&3 plot, p=0.03 and 0.07 for 1μM and 100nM MEHP, respectively (D) Scatter plots of principal component (PC) scores. For 10μM MEHP, p=0.048 in PC1&2 plot.

Table S1. Primer sequences used for qPCR

Gene	Forward (5' to 3')	Reverse (5' to 3')
18S rRNA	ATGGCCGTTCTTAGTTGGTG	TCTAAGGGCATCACAGACCT
MCP-1	CTCTCTTCCTCCACCACCAT	ACTGCATCTGGCTGAGCCA
TNF-α	TCTCATGCACCACCATCAAGGACT	TGACCACTCTCCCTTTGCAGAACT
CXCL1	ACCCAAACCGAAGTCATAGCC	TTGTCAGAAGCCAGCGTTCA
IL-6	TCCAGTTGCCTTCTTGGGACTGAT	AGCCTCCGACTTGTGAAGTGGTAT
PPARγ	GCACTGCCTATGAGCACTTCA	ACCATTGGGTCAGCTCTTGT
C/EBPα	AGACATCAGCGCCTACATCG	TGTAGGTGCATGGTGGTCTG
PEPCK	AGCGGATATGGTGGGAAC	GGTCTCCACTCCTTGTTC

Table S2. Discriminatory proteins from PLS-DA.

LV (-/+)[a]	Protein ID (gene name)	SwissProt accession #
LV1 (-)	Histone H4 (Hist1h4a)/	P62806
	Superoxide dismutase [Cu-Zn] (Sod1)	P08228
	Acyl-CoA-binding protein (Dbi)	P31786
	78 kDa glucose-regulated protein (Hspa5)	P20029
	Stress-70 protein, mitochondrial (Hspa9)	P38647
	Histone H2A type 2-A (Hist2h2aa1), Histone H2A type 1-H (Hist1h2a), Histone H2A.x(H2afx)	Q6GSS7, Q8CGP6, P27661
	Calmodulin (Calm1)	P62204
	Serpin H1 (Serpinh1)	P19324
	Malate dehydrogenase, mitochondrial (Mdh2)	P08249
	40S ribosomal protein S19 (Rps19)	Q9CZX8
	Voltage-dependent anion-selective channel protein 1 (Vdac1)	Q60932
	Peroxiredoxin-5, mitochondrial (Prdx5)	P99029
	Protein disulfide-isomerase (P4hb)	P09103
LV1 (+)	40S ribosomal protein S13 (Rps13)	P62301
	Pyruvate kinase PKM (Pkm)	P52480
	Annexin A1 (Anxa1)	P10107
LV2 (-)	Protein S100-A11 (S100a11)	P50543
	Tubulin beta-5 chain (Tubb5), Tubulin beta-6 chain (Tubb6), Tubulin beta-2A chain (Tubb2a), Tubulin beta-4B chain (Tubb4b)	P99024, Q922F4, Q7TMM9, P68372
	Hemoglobin subunit alpha (Hba)	P01942

	Alpha-actinin-4 (Actn4)	P57780
	Fatty acid synthase (Fasn)	P19096
	6-phosphofructokinase, liver type (Pfkl)	P12382
	NAD(P) transhydrogenase, mitochondrial (Nnt)	Q61941
	Fatty acid-binding protein, epidermal (Fabp5)	Q05816
	Stress-70 protein, mitochondrial (Hspa9)	P38647
	Core histone macro-H2A.1 (H2afy)	Q9QZQ8,
	6-phosphogluconolactonase (Pgls)	Q9CQ60
	ATP synthase subunit beta, mitochondrial (Atp5b)	P56480
	10 kDa heat shock protein, mitochondrial (Hspe1)	Q64433
	Alpha-enolase (Eno1)	P17182
	Glyceraldehyde-3-phosphate dehydrogenase (Gapdh)	P16858
LV2 (+)	GTP:AMP phosphotransferase AK3, mitochondrial (Ak3)	Q9WTP7
	Peroxisomal multifunctional enzyme type 2 (Hsd17b4)	P51660
	Actin, cytoplasmic 1 (Actb), Actin, aortic smooth muscle (Acta2), Actin, alpha skeletal muscle (Acta1)	P60710,<br>P62737,<br>P68134,
	60S acidic ribosomal protein P0 (Rplp0)	P14869
	Ran-specific GTPase-activating protein (Ranbp1)	P34022
LV3 (-)	Triosephosphate isomerase (Tpi1)	P17751
	1-acylglycerol-3-phosphate O-acyltransferase ABHD5 (Abhd5)	Q9DBL9
	60 kDa heat shock protein, mitochondrial (Hspd1)	P63038
	Serine/threonine-protein kinase WNK3 isoform 1 (Wnk1)	Q80XP9
	Glutamate dehydrogenase 1, mitochondrial (Glud1)	P26443

LV3 (+)	Vimentin (Vim)	P20152
	40S ribosomal protein S24 (Rps24)	P62849
	Rab GDP dissociation inhibitor beta (Gdi2)	Q61598
	Stress-70 protein, mitochondrial (Hspa9)	P38647
	Histone H2B type 1-B (Hist1h2bb)	Q64475
	Prostaglandin E synthase 3 (Ptges3)	Q9R0Q7
	Annexin A2 (Anxa2)	P07356
	Trafficking protein particle complex 9 (Trappc9)[b]	Q3U0M1
	Zinc finger FYVE domain-containing protein 16 (Zfyve16)[b]	Q80U44

[a] The far left column indicates whether the loading value was in the positive (+) or negative (-) latent variable (LV) space. Significant discriminatory proteins were identified for the first three LVs.

[b] One peptide found for the indicated protein. A protein identification was considered highly confident if at least two unique peptides were found at 99% confidence for that protein. All other discriminatory proteins in the table meet this criteria. 760 unique proteins were identified at a global 1% FDR. The full list of detected proteins and raw peptide data will be made available to interested readers upon request.
